# Willingness and attitudes of the general public towards the involvement of medical students in their healthcare

**DOI:** 10.1186/1753-6561-6-S4-P51

**Published:** 2012-07-09

**Authors:** H Shereef, M Abu Jubain, H Alobaidi, S Bholah, R Koghar, F Kanani

**Affiliations:** 1 University of Birmingham, UK

## Introduction

To determine if a correlation exists between the level of invasiveness of a clinical procedure and patient willingness for the procedure to be performed by medical students, and how this is affected by patient demographics and previous encounter(s). Previous research in the area has been on a small scale and have only looked at the passive involvement of medical students (i.e. in an observational capacity).

## Methods

We used a standardised questionnaire to conduct a cross sectional street survey in various areas of Birmingham, UK. Clinical procedures were categorised into two groups of increasing levels of invasiveness; history taking/examinations (comprising history taking, non-invasive and invasive examinations) and other clinical procedures (comprising measuring blood pressure, venepuncture and tube insertion (intubation)). Responders were asked to rank their willingness for medical students to perform each procedure.

## Results

We received a total of 293 responses. In both the history taking/examinations and other clinical procedures groups, willingness decreased with level of invasiveness, although the trend was less apparent in Whites and those aged 65+. Rates of willingness were significantly higher in females, the two older age groups (41-64, 65+) and Whites for history-taking; Whites and those aged 65+ for non-invasive examinations; the two older age groups (41-64, 65+) and Whites for invasive examinations; females, the two older age groups (41-64, 65+) and Whites for measuring blood pressure; and the two older age groups (41-64, 65+) and those who had previous encounter(s) for venepuncture. No significant associations were found for tube insertion (intubation).

## Conclusions

People in the community generally report that they are more willing to allow medical students to perform non-invasive procedures compared to more invasive procedures. This may limit opportunities for medical students to attain clinical competencies. An effective method of identifying more willing patients is needed, to benefit students while ensuring patient satisfaction in clinical settings.

